# Prognostic classification of endometrial cancer using a molecular approach based on a twelve-gene NGS panel

**DOI:** 10.1038/s41598-019-54624-x

**Published:** 2019-12-02

**Authors:** Raquel López-Reig, Antonio Fernández-Serra, Ignacio Romero, Cristina Zorrero, Carmen Illueca, Zaida García-Casado, Andrés Poveda, José Antonio López-Guerrero

**Affiliations:** 1 Laboratory of Molecular Biology, Services of Fundación Instituto Valenciano de Oncología, Valencia, Spain; 2 Medical Oncology, Fundación Instituto Valenciano de Oncología, Valencia, Spain; 3 Gynecology, Fundación Instituto Valenciano de Oncología, Valencia, Spain; 4 Pathology, Fundación Instituto Valenciano de Oncología, Valencia, Spain; 5 Department of Oncology, INITIA ONCOLOGY, Hospital Quirón Salud, Valencia, Spain

**Keywords:** Molecular medicine, Prognostic markers, Endometrial cancer

## Abstract

Endometrial Cancer (EC) is one of the most common malignancies in women in developed countries. Molecular characterization of different biotypes may improve clinical management of EC. The Cancer Genome Atlas (TCGA) project has revealed four prognostic EC subgroups: POLE, MSI; Copy Number Low (CNL) and Copy Number High (CNH). The goal of this study was to develop a method to classify tumors in any of the four EC prognostic groups using affordable molecular techniques. Ninety-six Formalin-Fixed Paraffin-embedded (FFPE) samples were sequenced following a NGS TruSeq Custom Amplicon low input (Illumina) protocol interrogating a multi-gene panel. MSI analysis was performed by fragment analysis using eight specific microsatellite markers. A Random Forest classification algorithm (RFA), considering NGS results, was developed to stratify EC patients into different prognostic groups. Our approach correctly classifies the EC patients into the four TCGA prognostic biotypes. The RFA assigned the samples to the CNH and CNL groups with an accuracy of 0.9753 (p < 0.001). The prognostic value of these groups was prospectively reproduced on our series both for Disease-Free Survival (p = 0.004) and Overall Survival (p = 0.030).Hence, with the molecular approach herein described, a precise and suitable tool that mimics the prognostic EC subtypes has been solved and validated. Procedure that might be introduced into routine diagnostic practices.

## Introduction

Endometrial Cancer (EC) is the most common gynecological neoplasm and the fourth most frequent cancer in women in developed countries, with 280000 cases per year worldwide^1^. This cancer principally affects post-menopausal women, with the peak incidence between 55 and 65 years^1^. Clinically, the presence of metrorrhagia in 80% of patients allows both early diagnosis and treatment, resulting in an improved five-year survival^2^. Among newly-diagnosed women, 68% will present localized disease in the uterine cavity, 20% will show disease in pelvic organs and lymph nodes, and about 8% will suffer distant metastasis at diagnosis^3^. Prognosis varies dramatically according to the stage of the disease. Stage I has an 80–90% five-year survival rate, whereas for Stage IV this rate decreases up to 20%^4,5^.

Considering the biology and clinical parameters, EC is classified into two groups: type I carcinomas comprise 80% of newly-diagnosed EC and are characterized by alterations in *PTEN*, *KRAS*, and *CTNNB1* and by microsatellite instability (MSI). These tumors are associated with better prognosis^6,7^. Type II tumors are defined by *TP53* mutations, high Ki-67 score, p16 inactivation and *CDH1* and *HER2* amplification^8,9^.

Integration of clinicopathological information and genetic data provides more accurate classification of EC into different prognostic groups, facilitating the use of specific therapeutic interventions. The integrated genomic characterization of EC performed by the Cancer Genome Atlas (TCGA) consortium^10^ defined four prognostic EC subgroups, with a prognosis from the best to the worst as follows: POLE group, comprising tumors with *POLE* exonuclease domain mutations; MSI group, composed of EC with MSI; Copy Number Low (CNL) and Copy Number High (CNH) groups. CN groups are defined by a differential profile of CN alterations (CNA), CNH group particularly presenting an elevated incidence of *TP53* alterations^10^.

The aim of this study was to develop a molecular prognostic classifier for EC that mimics the four TGCA prognostic groups, by using only a small multi-gene NGS panel and MSI determination.

## Results

### Selection of the multigene-NGS panel and mutational analysis

The EC data set from TCGA^10^ defines 48 genes with differential mutation frequencies across the four prognostic groups. A subset of 13 genes, corresponding to those with the highest differences in terms of frequencies between groups, was selected: *POLE*, *PTEN*, *TP53*, *ARID1A*, *KRAS*, *ARID5B*, *FBXW7*, *PPP2R1A*, *CTCF*, *CTNNB1*, *RPL22*, *PIK3CA*, *PIK3R1*. Two separate sequencing runs, containing 48 dual-pool libraries each were performed. The coverage, quality parameters and statistics were comparable between both runs, hence it was possible to merge the data for analysis. Sequencing metrics for analyzed samples are summarized in Supplementary File 1.

A median of 40 genetic alterations per case (range: 13–171) were found (Supplementary File 2). Variants were classified as mutated if they were already reported in ClinVar or if appeared as predicted pathogenic, likely pathogenic or VUS by PolyPhen and SIFT predictors. Benign and likely benign variants were not considered for the analysis. The presence of mutation was treated as categorical dichotomous variable (presence/absence of mutation).

**Figure 1:**
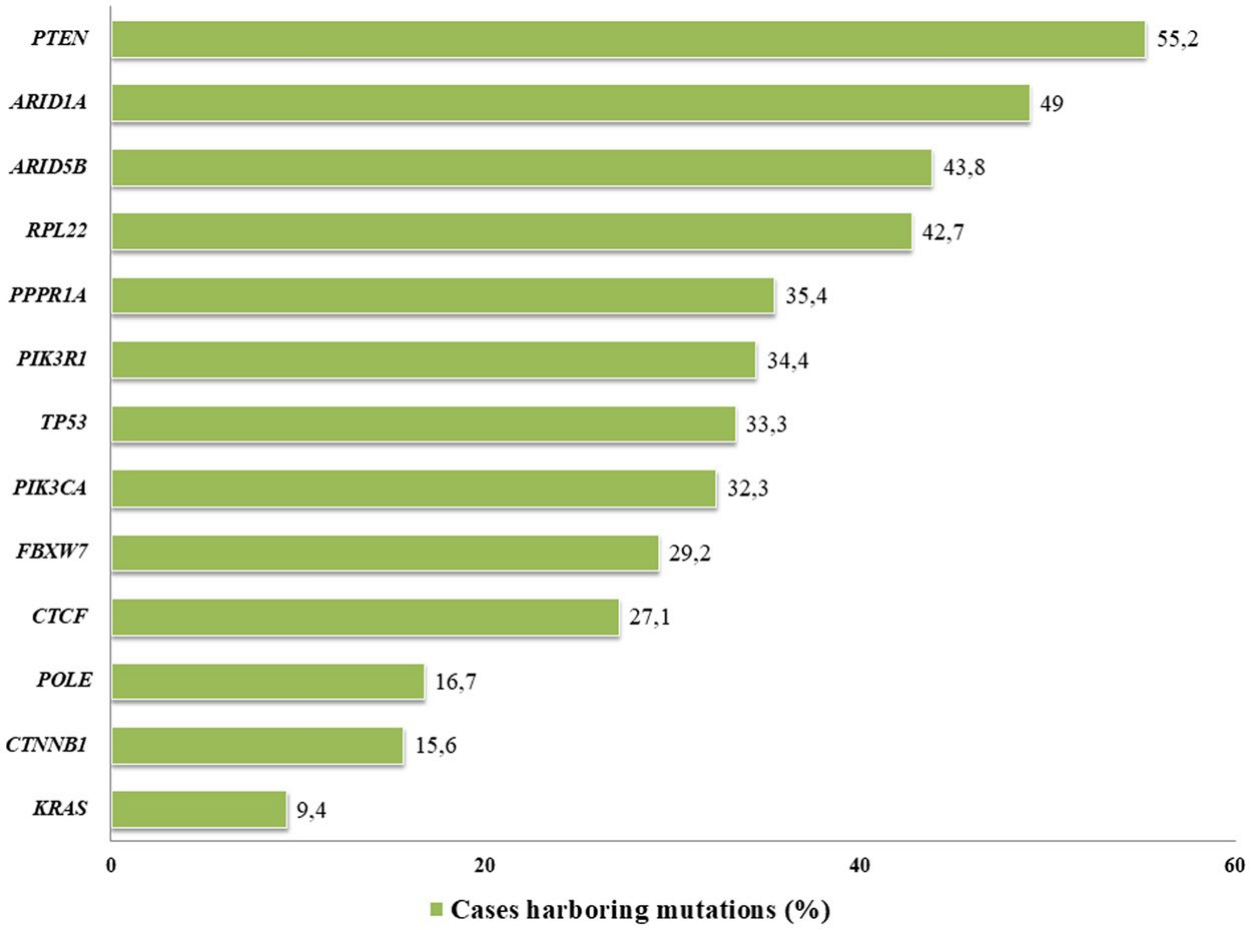
Frequency of gene mutations in EC patient’ series determined by NGS 13 genes panel. *Hotspot *POLE* (p.P286R and p.V411L) 5.2% (5.6% in TCGA population).

The most frequently affected genes in our series was *PTEN* (55.2%), followed by *ARID1A* (49.0%) and *ARID5B* (43.8%), whereas *KRAS* mutations (9.4%) represent the lowest frequency (Fig. 1). The median number of mutations per patient was 9.5 (range: 2–64). Univariate analysis at gene level showed a correlation between *POLE* mutation and early stage EC (p = 0.040), *PTEN* mutations were enriched in EC with endometrioid histology (p < 0.001) and low-grade tumors (p < 0.001). EC with serous histology harbored more *TP53* mutations (p = 0.021). Finally, *RPL22* mutation showed higher frequency in endometrioid histology (p = 0.005) and low-grade tumors (p = 0.004). *KRA*S (p = 0.035) and *CTCF* (p = 0.05) mutations were also related with low-grade tumors (Table 1A).

**Table 1:** Correlation between mutational status of analyzed genes and (A) Main clinical and pathological parameters in EC using Chi-square test (B) PFS and OS measured by log-rank test.

		Histology			Stage			Grade			
		Endometrioid	Serous	p-value	Early-stage	Advanced-stage	p-value	I	II	III	p-value
(**A**)											
POLE	mutated	15	1	N.S.	16	0	0.040	10	5	1	N.S.
	non-mutated	68	12		63	17		35	23	22	
PTEN	mutated	53	0	<0.001	44	9	N.S.	30	20	3	<0.001
	non-mutated	30	13		35	8		15	8	20	
TP53	mutated	24	8	0.021	24	8	N.S.	12	8	12	0.050
	non-mutated	59	5		55	9		33	20	11	
KRAS	mutated	8	1	N.S.	9	0	N.S.	8	0	1	0.035
	non-mutated	75	12		70	17		37	28	22	
CTCF	mutated	25	1	N.S.	22	4	N.S.	16	7	3	0.050
	non-mutated	58	12		57	13		29	21	20	
RPL22	mutated	40	1	0.005	34	7	N.S.	17	19	5	0.004
	non-mutated	43	12		45	10		28	9	18	
		**DFS**			**OS**						
		**Univariate**		**Multivariate**	**Univariate**		**Multivariate**				
(**B**)											
Stage	Early	37.40 (2.067–91.2)	0.006	N.S.	42.57 (2.067–91.20)	0.004	N.S.				
	Advanced	15.37 (4.87–91.00)			34.47 (6.30–91.00)						
Grade	I	50,33 (2.07–91.02)	0.003	N.S.	52.83 (2.067–91.20)	<0.001	8.26 (62.50–1.10 9	0.040			
	II	30.28 (9.70–79.57)			33.10 (9.70–79.57)						
	III	26.38 (4.87–67.60)			32.50 (6.30–67.60)						
Histology	Endometrioid	43.63 (5.47–37.40)	<0.001	8.90 (29.90–2.71)	<0.001	44.23 (2.067–91.20)	<0.001	N.S.			
	Serous	21.47 (2.067–91.20)				29.53 (6.30–38.6)					
TCGA groups	POLE	55.40 (24.27–77.43)	0.004		N.S.	55.40 (24.27–77.43)	0.030	N.S.			
	MSI	38.33 (11.9–74.93)				38.6 (11.9–74.93)					
	CNL	34.43 (2.067–91.00)				42.57 (2.067–91.00)					
	CNH	27.70 (4.87–91.2)				30.53 (6.30–91.2)					

Regarding the prognostic value of individual gene mutations in our series, mutations in *POLE*, *PTEN PIK3R1*, *ARID5B* and *PPP2R1A* are correlated with better patient outcome as seen in Supplementary File 3.

### Distribution of microsatellite instability in paired blood and FFPE samples

MSI was observed in 15 of 96 patients (15.6%): 14 of 15 with endometrioid histology (93.3%), and in just 1 of 13 serous cases (7.7%) (p = N.S). MSI was more frequent in early stages: 11/15 (73.3%) stages I-II vs. 4/14 (26.7%) stages III-IV (p = N.S). This parameter lacked prognostic value both for PFS and OS (Supplementary File 3). The status of Mismatch repair (MMR) proteins was also evaluated by immunochemistry (**IHC**, Supplementary Information) obtaining a concordance with MSI results of 96%.

### Building a predictive multi gene model using a Random Forest approach

A random forest (RF) predictive model for a dichotomous variable (CNL or CNH) was trained using the mutational profile of the 13 selected genes from 148 patients analyzed by the EC TCGA project^10^. To correctly adjust the RF model, the TCGA dataset was randomly split in two cohorts (training and validation), based on the distribution of the dichotomous response variable; hence, the groups consisted of 62 patients for the training set and 86 for the validation set.

To train the model, genotyping of 12 genes was included as categorical dichotomous variables (the so called 12g-model) (Tables 2 and 3). Prior to the adjustment of the RFA model, the number of variables per level on each split was optimized to pre-train the model. The model was validated with 5-fold cross-validation and bagging^11^.

**Table 2:** Contribution of evaluated parameters to 12 g-model measured as mean decrease of Gini index of the variables in the models.

Parameter	12 g-model
*TP53*	12.4658
*PTEN*	6.094
*CTNNB1*	3.4884
*ARID1A*	1.8658
*PPPR1A*	1.5958
*CTCF*	1.1435
*PIK3CA*	0.5644
*KRAS*	0.3994
*FBXW7*	0.4852
*PIK3R1*	0.4506
*ARID5B*	0.2425
*RPL22*	0

**Table 3:** Performance parameters of 12 g model.

	12 g-model RFA
Accuracy (95% CI)	0.9753 (0.9136–0.997)
No Information Rate	0.6049
Kappa	0.9483
McNemar’s test p-value	1
Sensitivity	0.9688
Specificity	0.9796
Positive Predictive Value	0.9688
Negative Predictive Value	0.9796
Prevalence	0.3951
Detection Rate	0.3827
Detection prevalence	0.3951
Balanced accuracy	0.9742

The POLE and MSI groups were directly defined by the presence of *POLE* mutations and MSI respectively.

### Impact of 12 genes RF model in the clinical stratification of the disease

Our series of 96 EC patients was stratified into the four TCGA prognostic groups based on the genotyping data of the 12-gene NGS panel, MSI status, grade, stage and histology: PO LE, 16/96 (16.7%); MSI-H, 12/96 (12.5%); CNH, 20/96 (20.8%); and CNL, 48/96 (50.0%). As mentioned above, CNH and CNL groups were classified with our RF adjusted model.

**Figure 2:**
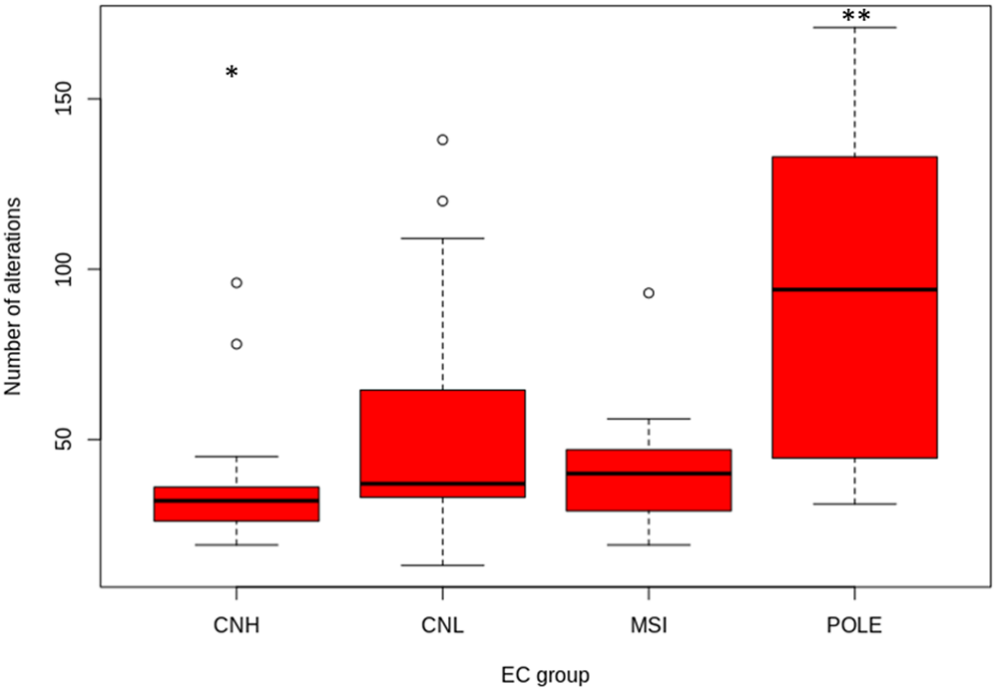
Mutational load across four EC prognostic subtypes. CNH group shows the lowest mutational rate (*p < 0.05), whereas POLE mutational rate is the highest (**p < 0.001).

**Figure 3:**
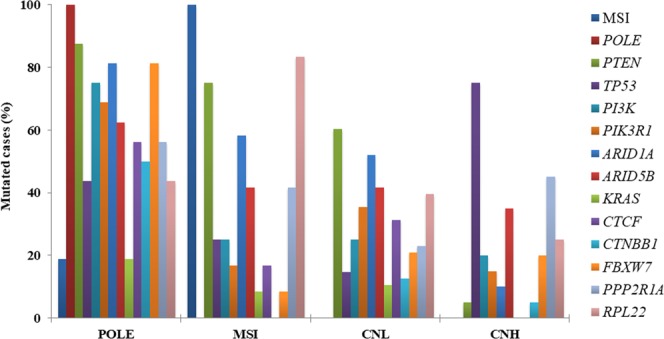
Distribution of genetic alteration across the four EC prognostic subtypes.

The POLE group was characterized by a *POLE* exonuclease domain mutation in all 16 cases and by the presence of MSI in 3 of the 16 cases (18.7%). This group presented the highest mutational ratio with a median of 94 variants/case (range: 31–171) compared with the other groups (p < 0.001) (Fig. 2). MSI group was characterized by the presence of microsatellite instability in 100% of the cases and had no POLE mutations. This group presented a lower median of alterations than POLE with 40 variants per case (range: 19–93). Among these alterations, the most affected genes were *PTEN* (75.0%), *ARID1A* (58.3%) and *RPL22* (83.3%). CNH presented a median of 32 variants per case (range: 19–96) and was characterized by mutations in *TP53* (75%), low frequency of *PTEN* mutations (5%) and alterations in *PPP2R1A* (45%). Finally, CNL showed a median of 37 variants per case (range: 13–138) (Table 4 and Supplementary Fig. S1). Gene by gene analysis of these alterations revealed that: *PTEN* (60.4%) and *TP53* (14.6%) presented the highest and the lowest mutation rate respectively, with alterations in other genes as follows: *PIK3R1* (35.4%), *ARID5B* (41.7%), *CTCF* (31.3%) and *RPL22* (39.6%). The distribution of mutations across groups in EC dataset is depicted in Fig. 3 (Supplementary Table S1).

**Table 4:** Occurrence of mutations sorted by functional annotation among EC prognostic subtypes (Median number of alteration/group).

	Molecular group (variants/group)			
	POLE	MSI	CNL	CNH
Regulator	11.0	5.7	6.9	4.8
**Frameshift**	**6**.**4**	**3**.**3**	**2**.**5**	**2**.**0**
In frame	3.3	2.9	2.8	2.0
Splicing events	9.3	5.1	4.6	3.8
Synonymous	24.5	13.1	14.5	9.5
Intron Variant	13.5	8.7	10.0	6.9
**Nonsense**	**3**.**9**	**0**.**8**	**0**.**8**	**0**.**5**
**Missense**	**35**.**2**	**11**.**4**	**10**.**5**	**9**.**0**

**Figure 4:**
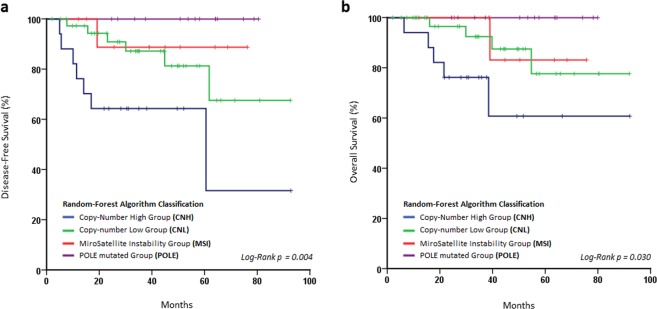
Kaplan-Meier plots assessed by log-rank test to evaluate. (**a**) Disease free survival and (**b**) Overall Survival according to 12 g stratification. Both parameters reach the statistical signification.

The Log-Rank test was used to evaluate the prognostic capacity of our molecular classification. This test confirmed that the molecular stratification of our patients revealed differences in both PFS (p = 0.004) and OS (p = 0.030), suggesting that the POLE and CNH biotypes constituted the best and the worst prognostic groups respectively, mirroring the groups defined by the TCGA (Fig. 4). In addition, a multivariate analysis was performed, being statistically significant only for histology (Table 1B).

## Discussion

One of the main problems in the management of EC patients is inter-observer agreement when assigning histology and tumor grade by microscopic techniques. These classifications are associated with different natural histories, treatment scheme and patient outcomes, all of which will influence clinical decision making. Thus, accurate pathological assessment of histology and grade is essential in prognosis assessment and patient management^12^. However, this scenario is frequently idealistic. For instance, a misclassification in grade assignment, especially in high-grade EC tumors, has already been reported^12^. In addition, there is a poor correlation of histology and grade between diagnostic and final tissue samples^13,14^. Moreover, inter-observer grade agreement has also shown only moderate consistency having a kappa index of 0.41–0.68^15^.

In the post-genomic era, multiomic information is redefining tumor classification. In this context, the EC TCGA project was developed and now constitutes an excellent source of data to mine prognostic models^10^. EC TCGA described four prognostic groups based on multiomics data. However, this approach is unaffordable in clinical scenarios due to the lack of availability of omic techniques in standard clinical laboratories. Several research groups have made great efforts to overcome these limitations. With this aim, different approaches have been applied to reproduce prognostic classification simplifying the methodology. Stello *et al*. used IHC for p53 and MMR protein assessment and Sanger sequencing for *POLE* hotspots genotyping as surrogate of the EC TCGA subgroups^16–18^. Similarly, the ProMisE/Vancouver group provided a molecular classification based on p53 IHC as a surrogate of the CNH/CNL TCGA groups^19,20^. However, the aforementioned inter-observer variability implicit in IHC makes standardization difficult between labs (Supplementary Fig. S2). This is underlined by the discrete (70%) concordance found in our global series, which is significantly improved in the CNH subgroup (84%), between the determination of *TP53* mutational status using IHC and NGS approach. For these reasons our objective was to develop a method based on the genotyping of only 12 genes with the definition and implementation of a reproducible RF model (12g-algorithm) to classify EC into the four prognostic groups.

We designed a small NGS gene panel with data from the EC TCGA dataset consisting of 13 of the most discriminant genes which presented the highest absolute and differential mutational frequency among the groups. The POLE ultramutated group was defined by mutations in the exonuclease domain of this gene. This group presents the highest mutational load and the best prognosis, as previously described by EC TCGA^10^. It should be highlighted that our cohort was significantly high in *POLE* mutations (16.7%) compared with the TCGA dataset (7%)^10^. This mismatch is presumably due to different NGS technical approaches implemented in the two projects. Whole Exome Sequencing (WES), used in the analysis of EC TCGA samples, achieved a lower coverage (20X) than the targeted panel which was implemented in our work (600X). Therefore, mutations with lower variant allelic frequency could not be detected in WES. However, the percentage of mutations found in POLE hotspots (p.P286R and p.V411L) defined by the EC TCGA^10^ was concordant between both datasets; 5.3% (our series) and 5.2% (TCGA series). Additionally, the MSI Group was obtained by the determination of MSI status using the eight microsatellite markers (NR27, NR21, NR24, BAT26, BAT25, D5S346, D2S123 and D17S250) by fragment analysis. The IHC for MMR proteins was also performed, obtaining a 96% of concordance between IHC and MSI results. This group was characterized by high mutational ratio, although lower than the POLE group (94 vs 40 median of mutations per case respectively).

The most challenging task was to define a surrogate to classify CNH and CNL groups, which currently requires sophisticated technology as well as technical and analytical training. To achieve this, we adjusted a RF model (12g-algorithm) by using the EC TCGA dataset. This 12 g model accurately defines CNH and CNL groups (97%) and considers the contribution of each gene to discriminate between groups. Finally, we validated the model with our prospective and independent EC patients series resulting in a total of 20 cases classified as CNH (21%) and 48 as CNL (50%). These frequencies were similar to those reported by the TCGA (26% and 39% respectively)^10^. As expected, these groups had lower mutational load than the POLE group and were characterized by mutations in *TP53* and *PTEN*. As a whole, this approach showed a good correlation with the TCGA groups and matched its prognostic value. In addition, our prognostic model classified the patients independently of IHC, thus avoiding the intrinsic inter-observer subjectivity.

Besides sequencing and adjusting the 12 g-RF model, we trained another model including clinicopathological features (histology, grade and stage) to study the influence of these parameters, the so-called CPP model.

Although there was a slight improvement in the performance parameters of the RFA (Supplementary Tables S2 and S3), it is important to take into account that our series came from a monographic oncology hospital. Additionally, pathological assessment was performed by a single gynecological pathologist highly trained in the diagnosis of EC, possibly masking the subjective effect.

Our approach overcomes subjectivity and technical difficulties related to the definition of CNH and CNL groups. The assessment of the mutational status by NGS technology constitutes a highly objective methodology, drastically simplifying the approach. Furthermore, the common availability of NGS and trained staff in clinical labs will facilitate the implementation of the proposed workflow in the diagnostic routine.

In conclusion, we have defined a prognostic model to classify EC prognostic biotypes based on the analysis of a multi-gene NGS panel; which could be easily implemented as a molecular diagnostic tool.

## Material and Methods

### Patients

This study includes 96 EC patients prospectively collected from 2010 to 2019 within the context of the institutional projects ACOG0901 and ACOG1602. Experimental protocols were approved by Instituto Valenciano de Oncología (IVO) Institutional Review Board in 2009 and 2016 respectively. All methods used during the study were performed in accordance with the relevant guidelines and regulations.

At the time of the study, our prospective institutional EC database contained a total of 187 patients. Criteria for inclusion in this study was: age over 18 years; tumors with serous or endometrioid histology; grade I to III and stage I, II and III. A total of 149 fulfilled these criteria, from which 96 were selected according to the best ranked DNA quality and concentration.

All analyzed samples were formalin-fixed paraffin-embedded (FFPE) tumor tissue retrieved from the IVO Biobank. Informed consent of patients was obtained in accordance with our institution’s ethical and legal regulations.

Clinical and pathological information for the whole series was integrated into a prospective database, median age at diagnosis being 62 years (range: 36.4–87) and median follow-up of 35.02 months (range: 2.1–91.2 months). During follow-up, 15.2% of the patients recurred, and 10.7% died as consequence of the disease; the median progression-free survival (PFS) being 33.65 months (range: 2.1–91.2) and the median overall survival (OS) 35 months (range: 2.1–91.2 months) (Table 5).

**Table 5:** Distribution of patients based on most relevant clinical and pathological parameters in (A) TCGA series, (B) Our series.

Stage	Endometrioid			Serous	
	Grade 1	Grade 2	Grade 3	All	Total
(**A**)					
I	78 (23)	83 (24)	70 (21)	17 (5)	248 (73)
II	3 (1)	9 (3)	6 (2)	5 (1)	23 (7)
III	7 (2)	12 (4)	26 (8)	25 (7)	70 (21)
**Adjuvant therapy**					
RT	12 (3)	28 (8)	22 (6)	7 (2)	69 (19)
Chemo	2 (1)	6 (2)	14 (4)	13 (4)	35 (10)
ChemoRT	2 (1)	9 (3)	18 (5)	17 (5)	46 (13)
Unknown	70 (20)	61 (17)	57 (16)	16 (5)	204 (58)
Total	86 (24)	104 (29)	111 (31)	53 (15)	354 (100)
(**B**)					
I	40 (42)	24 (25)	6 (6)	7 (7)	77 (80)
II	0 (0)	1 (1)	0 (0)	1 (1)	2 (2)
III	5 (5)	3 (3)	4 (4)	5 (5)	17 (18)
**Adjuvant therapy**					
RT	21 (22)	8 (8)	2 (2)	1 (1)	32 (33)
Chemo	2 (2)	1 (1)	2 (2)	7 (7)	12 (13)
ChemoRT	3 (3)	3 (3)	6 (6)	3 (3)	15 (16)
Unknown	2 (2)	1 (1)	0 (0)	2 (2)	5 (5)
Total	28 (29)	13 (13)	10 (10)	13 (13)	64 (67)
**32** (**33**) **patients did not receive any treatment**					
Median follow-up (months)	34.45 (1.8–91.2)				
Median PFS (months)	33.1 (1.87–91.2)				
Median OS (months)	34.45 (1.87–91.2)				
Relapse (%)	14.6				
Exitus (%)	11.4				

### Multi-gene next generation sequencing

DNA extraction was performed using the QIAmp DNA FFPE Tissue kit (Qiagen, Valencia, CA) following the manufacturer’s instructions. Three FFPE blocks sections of 20 µm-thin with tumor content higher than 50% were used. The final DNA concentration was measured fluorometrically using PicoGreen™ reagent in a Quantifluor instrument (Promega, Fitchburg, Wisconsin). DNA sample quality for NGS selection was estimated using a qPCR-based approach (QC illumina kit) (Illumina, San Diego, CA). In addition, quality and related size of genomic DNA were assessed by the microfluidics-based platform Agilent 4200 Tapestation with Genomic D1000 Kit (Agilent, Santa Clara, CA). Electropherograms were visualized with the TapeStation Software Analysis A.02.01 SR1 including data collection, peak detection, and interpretation of the different profiles.

For NGS, the median starting DNA concentration was 49.91 ng/μl (8.77–189.538 ng/μl). According to the manufacturer’s protocol, the initial amount of DNA required to construct the library is between 10 and 100 ng. In some cases, recommended DNA quantity was not achieved, so maximum available quantity was assigned to these samples. Library preparation was conducted using TruSeq Custom Amplicon Low Input Kit (Illumina, San Diego, CA) in combination with a custom-designed panel (DesignStudio, Illumina, San Diego, California), interrogating the whole coding regions of the following 13 genes: *POLE*, *PTEN*, *TP53*, *ARID1A*, *ARID5B*, *FBXW7*, *PPP2R1A*, *CTCF*, *CTNNB1*, *RPL22*, *KRAS*, *PIK3CA*, *PIK3R1*. These genes were selected based on the sequencing results of the TCGA. By selecting the 13 genes that best discriminate between the 4 groups, based on relative and absolute frequency of each gene among the groups, it is possible to improve the feasibility of the model. Samples were subjected to dual-pool amplicon-based PCR library preparation according to the manufacturer’s instructions. Subsequent sequencing of pooled libraries was performed in a NextSeq. 550 sequencing platform (Illumina, San Diego, California).

Data analysis, including alignment to the hg19 human reference genome and variant calling, was done using CASAVA pipeline (Illumina, San Diego, CA). These variants were then annotated using the Illumina VariantStudio v3.0 data analysis software (Illumina, San Diego, CA). Integrative Genomic Viewer (Broad Institute) was used to visualize the sequence and check for the presence of mutations^21,22^. Variants were selected based on a minimum coverage of 600X, minimum frequency of mutated allele of 5% and previously describe or *in silico* as pathogenic, likely pathogenic or variant of unknown significance (VUS).

### Microsatellite instability

MSI was performed on 2–3 ng of DNA from paired FFPE and blood samples using the Type-it Mutation Detect PCR Kit (Qiagen) in a Veriti thermocycler (Applied Biosystem, Foster City, CA) and specific primers labelled with the fluorophores FAM, HEX or NED for the following STR regions: NR27, NR21, NR24, BAT26, BAT25, D5S346, D2S123 and D17S250^23^. PCR conditions were: 5′ initial denaturing at 95 °C followed by 35 cycles at 95 °C of 30″, 1′30″ at 60 °C and 30″ at 72 °C with a final 10′ extension at 68 °C. PCR products were denatured with formamide for 5′ at 95 °C and visualized, after capillary electrophoresis in the ABI3130xl Genetic Analyzer (Applied Biosystem, Foster City, CA), using the GeneMapper v4.0 software (Applied Biosystem, Foster City, CA). MSI-High (MSI-H) was considered when at least 30% of STR regions presented an MSI pattern.

### Random forest algorithm (RFA)

The EC dataset from TCGA^10^ was used to train a Random Forest algorithm (RFA) to define a prognostic model. Dichotomous and categorical variables including mutational status of the studied genes and clinical and pathological parameters such as histology, stage and grade were implemented in the model. Furthermore, a standard bagging approach is applied. Briefly, the dataset is internally split in three sets in order to internally cross-validate the predictor’s performance. The number of trees was empirically estimated to 1000. R v3.4.3 patched was used in all the predictive models built and tested.

### Survival analysis

Statistical analysis was performed to define the correlations between clinicopathological and molecular parameters for time-to-event variables [i.e., PFS and OS]. Log-rank test with Kaplan–Meier estimations were performed to compare groups. SPSS v20.0 software was used for statistics.

For categorical variables frequency inference a chi-square test was employed. For median comparison between continuous variables non-parametric tests (Kruskal-Wallis and Wilcoxon) were used.

For RFA classification validation, survival analysis of the four established groups was performed using log-rank test.

## Supplementary information

Supplementary Information

Supplementary file 1

Supplementary file 2

Supplementary file 3

## Data Availability

All data generated or analysed during this study are included in this published article (and its Supplementary Information files).
