# Supplementary material for: Prognostic classification of endometrial cancer using a molecular approach based on a twelve-gene NGS panel
Source: Sci Rep. 2019 Dec 2;9:18093. doi: 10.1038/s41598-019-54624-x (PMC6889294; doi:10.1038/s41598-019-54624-x)
Supplement: Supplementary file 1 — Supplementary Information [file 41598_2019_54624_MOESM1_ESM.pdf]

SUPPLEMENTARY INFORMATION

Prognostic classification of endometrial cancer using a molecular approach based on a twelve-gene NGS panel

Raquel López-Reig[1] , Antonio Fernández-Serra[1] , Ignacio Romero[2] , Cristina Zorrero[3] , Carmen Illueca[4] , Zaida García-Casado[1] , Andrés Poveda[5] , José Antonio López-Guerrero[1,].

[1]Laboratory of Molecular Biology, and Services of [2]Medical Oncology, [3]Gynecology and [4]Pathology, Fundación Instituto Valenciano de Oncología, Valencia, Spain. [5]Department of Oncology, INITIA ONCOLOGY, Hospital Quirón Salud, Valencia, Spain.

SUPPLEMENTARY METHODS

Immunohistochemistry analysis

The IHC study was performed in whole sections from formalin-fixed paraffin-embedded tissues. Microscopic slides (hematoxylin and eosin, H&E) from the selected cases were reviewed and confirmed by gynecology pathologist.

To IHC analysis, Antigen retrieval was performed by pressure cooker boiling at 1.2 atmospheres for 3 min in 10 μmol/L citrate buffer (pH 6.0). The LSAB method (Dako) was performed, followed by revelation with 3,30-diaminobenzidine as conventional protocols. Immunoreactivity was defined as negative, when there was no staining and positive when the staining was observed, except for p53 which staining was interpreted as normal (1-70%) or aberrant (0 or >70%)

The following panel of markers from Dako was used to evaluate the expression of proteins.

**Protein**	**Dilution**	**Clone**	**Manufacturer**
**MLH1**	Prepared to use	IRO79	DAKO
**PMS2**	Prepared to use	EP51	DAKO
**MSH2**	Prepared to use	FE11	DAKO
**MSH6**	Prepared to use	EP49	DAKO
**TP53**	Prepared to use	DO-7	DAKO

SUPPLEMENTARY TABLES AND FIGURES

Supplementary Table S1: Distribution of genetic alteration across the four prognostic groups.

	**Group**			
**Parameter**	**POLE**	**MSI**	**CNL**	**CNH**
**MSI (%)**	3 (18.7)	12 (100)	0 (0)	0 (0)
**POLE (%)**	16 (100)	0 (0)	0 (0)	0 (0)
**PTEN (%)**	14 (87.5)	9 (75)	29 (60.4)	1 (5)
**TP53 (%)**	7 (43.7)	3 (25)	7 (14.6)	15 (75)
**PI3K (%)**	12 (75.0)	3 (25)	12 (25.0)	4 (20)
**PIK3R1 (%)**	11 (68.7)	2 (16.7)	17 (35.4)	3 (15)
**ARID1A (%)**	13 (81.2)	7 (58.3)	25 (52.1)	2 (10)
**ARID5B (%)**	10 (62.5)	5 (41.7)	20 (41.7)	7 (35)
**KRAS (%)**	3 (18.7)	1 (2.0)	5 (10.4)	0 (0)
**CTCF (%)**	9 (56.2)	2 (16.7)	15 (31.3)	0 (0)
**CTNNB1 (%)**	8 (50)	0 (0)	6 (12.5)	1 (5)
**FBXW7 (%)**	13 (81.2)	1 (2.0)	10 (20.8)	4 (20)
**PPP2R1A (%)**	9 (56.2)	5 (41.7)	11 (22.9)	9 (45)

**RPL22 (%)**	7 (43.7)	10 (83.3)	19 (39.6)	5 (25)

Supplementary Table S2: Contribution of each parameter in the CPP model measured as decreasing of Gini index.

**Parameter**	**CPP-model**
TP53	**8.765**
**Grade**	**7.3384**
**Histology**	**4.7434**
PTEN	**4.2490**
CTNNB1	**1.6679**
ARID1A	**1.4816**
**Stage**	**0.7857**
PPPR1A	**0.6392**
CTCF	**0.5489**
PIK3CA	**0.4308**
KRAS	**0.4176**
FBXW7	**0.3973**
PIK3R1	**0.3395**
ARID5B	**0.2071**
RPL22	**0**

Supplementary Table S3: Performance description of the RF model including the 3 clinical and pathological parameters (grade, histology and stage)

	**CPP-model RFA**
**Accuracy (95% CI)**	**0.9808 (0.8974-0.9995)**
**No Information Rate**	**0.6923**
**Kappa**	**0.9541**
**McNemar's test p-value**	**1**
**Sensitivity**	**0.9375**
**Specificity**	**1**
**Positive Predictive Value**	**1**
**Negative Predictive Value**	**0.9730**
**Prevalence**	**0.3077**
**Detection Rate**	**0.2855**
**Detection prevalence**	**0.2855**
**Balanced accuracy**	**0.9688**

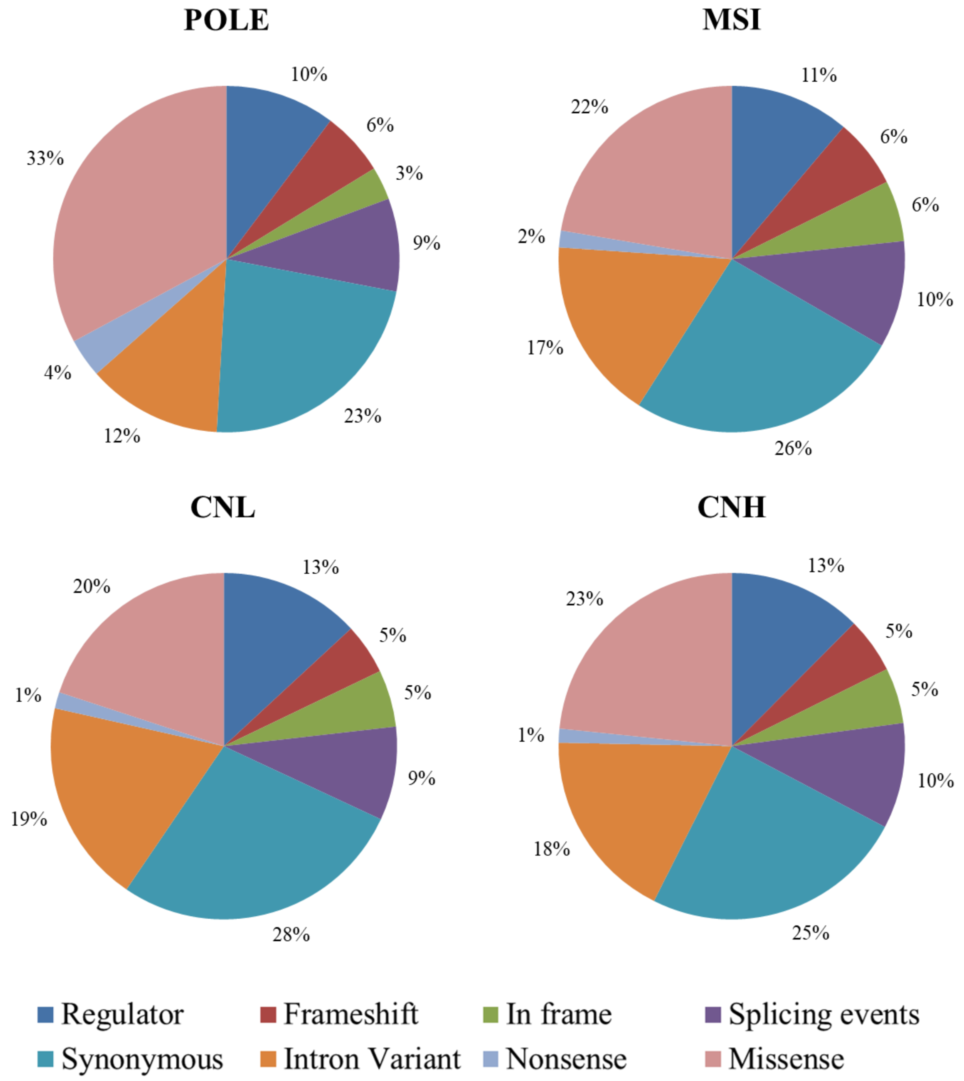

Supplementary figure S1: Distribution of alterations due to functional type among four EC prognostic groups.

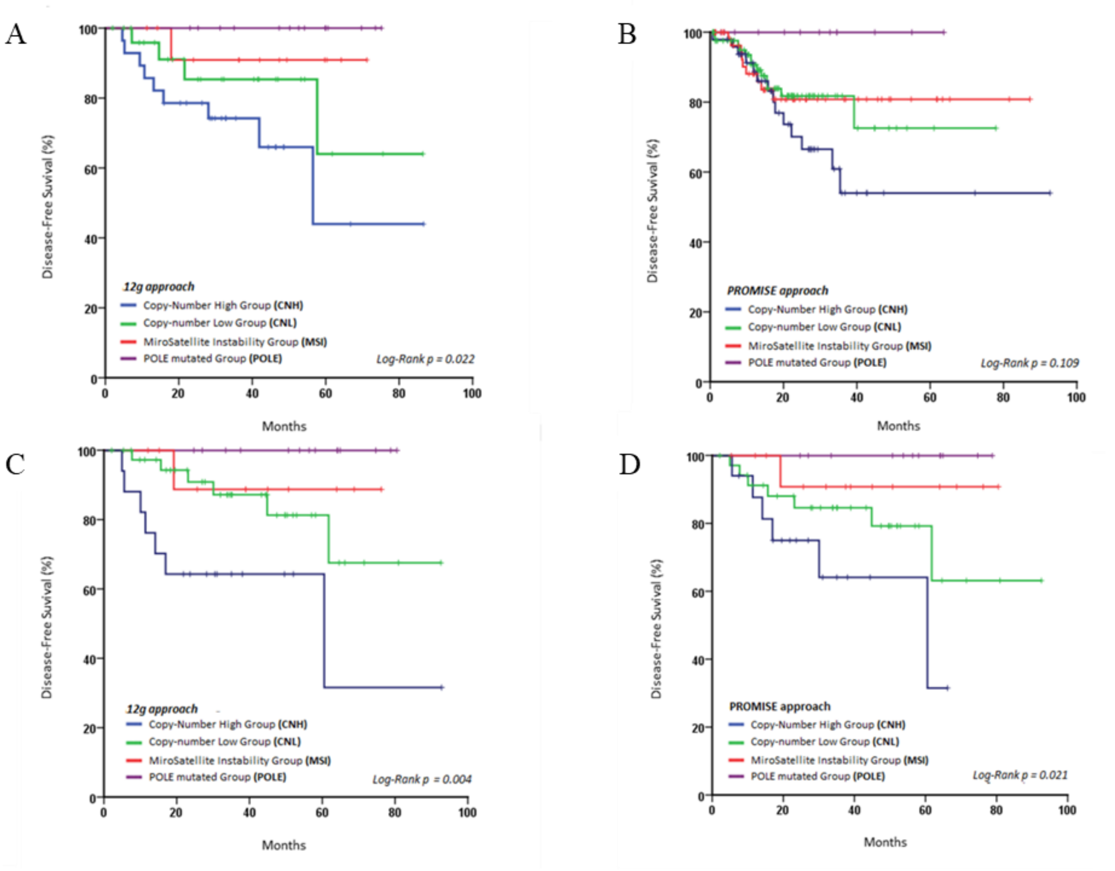

Supplementary Figure S2: Kaplan-Meier plots assessed by log-rank test to evaluate a) Disease free survival based on 12g classification approach in the EC-ATLAS series b) Disease free survival based on PROMISE classification approach in the EC-ATLAS series. a) Disease free survival based on 12g classification approach in our series b) Disease free survival based on PROMISE classification approach in our series. *PROMISE classification was inferred, in our series, based on MMR and TP53 IHC and *POLE* sequencing. However, ATLAS series, due to lacking of IHC information, was classified using POLE and *TP53* sequencing data and MSI analysis.
